# Thanos: An R Package for the Gene-Centric Analysis of Functional Potential in Metagenomic Samples

**DOI:** 10.3390/microorganisms12071264

**Published:** 2024-06-21

**Authors:** Zhe Zhao, Federico Marotta, Min Wu

**Affiliations:** 1 College of Life Sciences, Zhejiang University, Hangzhou 310058, China; zhaozhe@zju.edu.cn; 2 Structural and Computational Biology Unit, European Molecular Biology Laboratory, 69117 Heidelberg, Germany; federico.marotta@embl.de

**Keywords:** metagenomics, Rstats, KEGG, functional profiling, sequencing depth

## Abstract

As the amount of metagenomic sequencing continues to increase, there is a growing need for tools that help biologists make sense of the data. Specifically, researchers are often interested in the potential of a microbial community to carry out a metabolic reaction, but this analysis requires knitting together multiple software tools into a complex pipeline. Thanos offers a user-friendly R package designed for the pathway-centric analysis and visualization of the functions encoded within metagenomic samples. It allows researchers to go beyond taxonomic profiles and find out, quantitatively, which pathways are prevalent in an environment, as well as comparing different environments in terms of their functional potential. The analysis is based on the sequencing depth of the genes of interest, either in the metagenome-assembled genomes (MAGs) or in the assembled reads (contigs), using a normalization strategy that enables comparison across samples. The package can import the data from multiple formats and offers functions for the visualization of the results as bar plots of the functional profile, box plots of compare functions across samples, and annotated pathway graphs. By streamlining the analysis of the functional potential encoded in microbial communities, Thanos can enable impactful discoveries in all the fields touched by metagenomics, from human health to the environmental sciences.

## 1. Introduction

The field of metagenomics has experienced significant growth over recent decades [1], offering unprecedented insights into microbial communities across various environments, ranging from the human body [2,3,4] to marine ecosystems [5]. The progress was largely enabled by the advent of next-generation sequencing technologies and the development of algorithms for the reconstruction of individual microbial genomes from the pooled and fragmented DNA extracted from environmental samples [1,6]. As is often the case, however, collecting more data does not necessarily lead to an improved understanding of the underlying biological systems. Thus, with increasing sequencing data, there also increases the need for tools capable of interpreting them.

One of the most basic questions that can be asked concerns which taxa are present in a sample [7]. The phyloseq [8] package is a powerful tool for the exploration of microbiome profiles, offering a practical approach to investigating the taxonomic composition of metagenomic samples collected from diverse environments. However, the taxonomic profile offers only one view into the complex and multifaceted nature of biological samples. The gene composition of a sample offers a complementary view, one that can help answer questions such as: does carbon fixation occur in this environment? or: is methane metabolism more active in lakes or in the Atlantic ocean? This sort of information is not always reflected in the taxonomic profiles [9,10]. Furthermore, taxonomic profiling requires either reconstructing the full metagenome-assembled genomes (MAGs), which leads to a “binning bias” because unbinned sequences are not analyzed, or at least scanning for marker genes to recover the operational taxonomic units (OTUs) [11], which can be time-consuming.

As the functional profiling task is somewhat more complex than taxonomic profiling, there is currently no standard way to perform this analysis. Tools like Prokka [12,13] or the EggNOG-mapper [14,15] can indeed provide a bulk-level overview of the functional composition by performing sequence-similarity searches for each gene in the sample, but researchers are often interested in more specific questions about individual genes or metabolic pathways, and require a more in-depth analysis [10].

In this work, we present Thanos, an R package that offers a convenient way to perform functional profiling with a gene- or pathway-centric approach. The package provides quantitative functional information through a “depth score” for each gene of interest across samples: genes with a higher sequencing depth are assumed to be more prevalent and active in the sample. Moreover, we introduce a normalization strategy that makes depth scores comparable across samples and even across independent sequencing projects, enabling the comparative analysis of multiple environments at the same time. The depth scores of individual genes can also be aggregated into their natural higher-level units, the metabolic pathways imported from KEGG [16,17].

## 2. Materials and Methods

As R is one of the most popular languages for bioinformatics, not least thanks to the Bioconductor project [18], Thanos was implemented in R and makes use of its ecosystem. The overall design of the package draws inspiration from phyloseq [8]. Briefly, the main component of a phyloseq object is the OTU abundance table, in the form of a numeric matrix with taxa on the rows and samples on the columns (or vice versa). The OTU table can be optionally decorated with sample metadata, an expanded taxonomy table, a phylogenetic tree, and even the reference genome of each taxon. Naturally, phyloseq also provides functions to perform common manipulations on the abundance table, such as filtering samples, pruning taxa, or aggregating abundances by taxonomy. The main idea behind Thanos is that the same objects and methods can be used on gene abundances as well as taxa abundances. Thus, the main purpose of our package is to perform a mapping from the taxonomy space to the functional space of a metagenomic sample, while using phyloseq objects to keep track of the abundances. We therefore inherit all of the useful methods that have already been implemented in phyloseq.

We consider a sample to be a set of DNA sequences. As Thanos does not require (but can still make use of) the taxonomy information, the sequences can be either assembled contigs or binned MAGs. Each contig or MAG is associated with a number representing its sequencing depth in each sample and is initially stored in a phyloseq object. In order to map the sample to its functional space, Thanos uses the HMMER software [19] to perform a sequence similarity search. However, unlike existing tools such as Prokka [12,13] or the EggNOG-mapper [14,15], which search each gene in the sample against a database of target sequences, Thanos reverses this process, using the genes of interest as query and the sample as target database. The advantage of this approach is that it lets researchers build a custom profile hidden Markov model (HMM) for their gene of interest, allowing them to capture the specific sequence variation that they are interested in. Nevertheless, as custom profiles are not always necessary, Thanos also has the ability to automatically build a profile for a given gene by leveraging the KEGG orthologs database [16,17].

Once the profiles for the genes of interest have been generated, Thanos searches for them across the samples, keeping track of the contigs/MAGs where each gene is found, and summing their depths. Next, Thanos performs another HMMER search, this time not for a gene of interest, but for a control gene: a universal marker gene that is conserved across all microorganisms (bacteria, archaea, or both, depending on the research question). Dividing the total depth of a gene of interest by the total depth of the control gene within each sample produces a normalized score that can be interpreted as the average copy-number of the gene of interest in the sample, and can therefore be compared across independent samples. If taxonomic information is available, the depth calculation can be done for each taxon independently. The results are compiled into another phyloseq matrix, where instead of OTUs we have the gene or pathway of interest.

**Figure 1:**
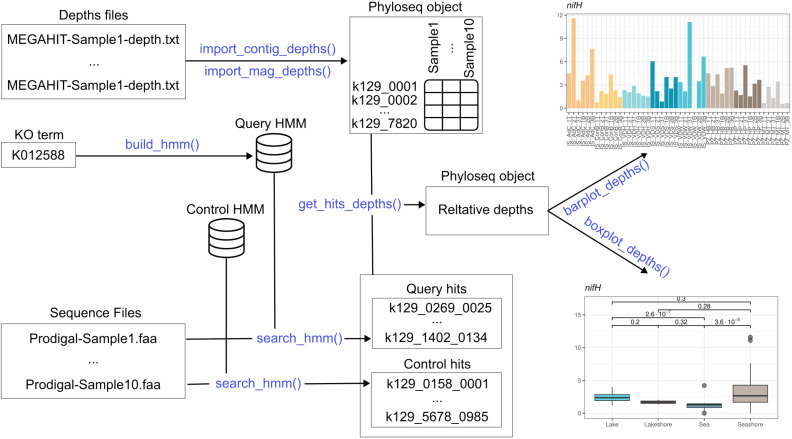
Overview of the Thanos workflow.

Finally, Thanos provides functions to visualize the results using the popular ggplot2 package [20]. There are three types of plot: bar plots, which show the depth profile across samples, potentially grouped by taxon; box plots, displaying aggregate statistics about groups of samples; and annotated reaction graphs, reproducing a KEGG module and coloring the reactions by the depths of the enzymes that catalyze them [16]. Figure 1 gives a global overview of the package’s functionality; in the subsequent subsections, the implementation will be discussed in detail.

### 2.1. Importing Depths Files

First, we need to import the pre-computed depths. These files can be obtained from standard tools in the metagenomic arsenal, such as MetaBAT [21,22] or CoverM [23], and are generated as part of a standard workflow [6]. Thanos can read depths files in these standard formats. In the case of MAG depths, there is typically a single file with all of the MAGs in the rows and the samples on the columns, and it can be imported directly as a phyloseq object. For contig depths, due to their large size, there is usually one file for each sample, where contigs from that sample are measured across all other samples; Thanos simply takes the list of files and concatenates them into a single phyloseq object. One complication is that the header of the contig depths files often has a prefix or suffix denoting the sample where the contig comes from; since each file would have a different header, this prevents the concatenation. For this reason the user has to specify a pattern and a replacement that will be applied to the headers, so that the sample-specific part can be removed.

### 2.2. Running HMMER

Next, we need a profile HMM file for each gene of interest. Typcal genes of interest are enzymes that perform key metabolic functions. If the user has already produced or downloaded custom HMMs, nothing else is needed. Otherwise, HMMs can be generated automatically from KEGG orthologs: the user just needs to supply a KO identifier, and all the genes in the orthologous family are downloaded, aligned, and converted into an HMM profile. The download uses the KEGGREST Bioconductor package, which interfaces with the KEGG API. For the alignment, we rely on the msa package from Bioconductor, which offers several choices for the algorithm, with “Muscle” being the default. Once the alignment is obtained, it is converted into an HMM profile using the HMMER software.

No matter how they were generated, the HMM files for the genes of interest must then be searched in the samples. For this purpose, the user is expected to provide sequence files in FASTA format containing the called genes from each entry in the previously generated phyloseq depths object. In particular, when dealing with MAG depths, there should be one FASTA file for each MAG, and when dealing with contig depths, there should be one FASTA file for each sample. These files are produced by popular tools like Prodigal [24,25] or Prokka [12,13], which are already included in comprehensive metagenomic pipelines like nf-core/mag [26,27,28]. Thanos provides the function search_hmm(hmm_file, target_fasta_files), which spawns an hmmsearch process and parses the results. In this case, the results will be the IDs of the genes that bear sequence similarity with the HMM profile, along with their respective scores. Users can specify the minimum score threshold to retain the hits. The search should be repeated for the control gene, for which Thanos already provides an HMM file, but users can also use their own if they wish. The default control gene is GrpE, a nucleotide exchange factor that is important for protein folding and heat-shock response  [29,30]. The HMM profile for this gene was derived from GTDB v214 [31,32] marker files: bac120_r214_reps_PF01025.20.afa.

### 2.3. Aggregation and Normalization

At the end of the search phase, we thus have a list of genes that are homologous to the given HMM profile, as well as a list of genes that are homologous to the control HMM profile. Since each sample usually contains many MAGs or contigs, and each individual MAG or contig contains many genes, there must be a mechanism to associate each gene to the MAG or contig where it comes from. Thanos uses a linker function, which can also be specified by the user, to achieve this. The linker function takes the name of a FASTA file and the ID of a gene, and returns the MAG or contig where it comes from. Through the linker function, it becomes possible to filter the MAGs or contigs according to whether they contain the target gene. For each sample, Thanos aggregates the depths of all the MAGs or contigs that contain the gene of interest and divides it by the aggregated depth of all the MAGs or contigs that contain the control gene. The resulting score represents how prevalent is the gene of interest compared to a universal single copy gene in each sample. For the convenience of the user, two linker functions that cover the most common cases are already built in. As an additional feature, when the taxonomy assignments of the MAGs are known, it is possible to stratify the score by taxonomy. This means that there will be one score for each taxon in each sample, making it easy to make hypotheses about the role of a particular taxon in the ecosystem. All the scores are saved in a phyloseq object, which is returned to the user.

### 2.4. Visualization

The last part of the Thanos workflow consists of the visualization of the results. Advanced users can, of course, compose their own plots starting from the aggregated results, but three plot types are also provided by default. The first is a bar plot of the depth scores. We provide a flexible interface where users can choose what to show on the *x*-axis, and the depths will be automatically aggregated by that variable. Indeed, as the results are normal phyloseq objects, they can be decorated with sample metadata or taxonomy tables. By default, samples are on the *x*-axis, but users may choose to aggregate the samples into subgroups, or to show the depths by taxonomy instead. The second plot type is a box plot, useful to compare groups of samples. Again, users have all the freedom to customize the groups. Finally, we provide a function that plots the KEGG reaction graph of a whole module, where each enzyme is colored by its depth score in the samples of interest.

### 2.5. Parallelization

As metagenomic datasets can be rather big, Thanos makes it possible to run the HMM searches in parallel, which can dramatically speed up the code execution. There are two nested levels of parallelism: first, users can control how many parallel hmmsearch processes are spawned, and second, for each process. it is possible to choose how many threads it will use. The outermost level of parallelism is most useful when there are many protein sequence databases, whereas the innermost level is especially useful when the individual sequence databases are large. Reading the contigs depths files can also be parallelized.

### 2.6. Dependencies

Thanos depends on R (version >= 4.3) [33] and the following packages: phyloseq (version >= 1.46), data.table (version >= 1.14), KEGGREST (version >= 1.42), msa (version >= 1.34), Biostrings (version >= 2.70), and ggplot2 (version >= 3.4). In addition, the HMMER (version >= 3.3) binaries must be installed separately.

## 3. Results

To illustrate the functionality of our software, we will showcase two applications. The data come from the TARA ocean project [34] and were already available in the European Nucleotide Archive (ENA) [35] under accession number PRJEB402. First, we downloaded the raw reads for two ocean “provinces” (according to the nomenclature of the original publication) that we selected: the Red Sea and the Mediterranean Sea, corresponding to 54 sequencing runs from 31 samples from 11 distinct stations. Then we used the nf-core/mag v2.5.4 automated pipeline for assembly, binning, and annotation [26]. As some samples were sequenced multiple times, we co-assembled all the reads coming from the same sample. The Experiment IDs of the sequencing runs and the co-assembly groups are summarized in Supplementary Table S1. Running the nf-core/mag pipeline provided all the files necessary for running Thanos.

### 3.1. MAGs Workflow: Sulfur Metabolism by Taxonomy

Our aim was to investigate which taxa have the potential to perform sulfur metabolism in these two seas. Thanos minimally requires three inputs: a list of genes of interest, the MAG depth files, and the protein sequence files, all generated by the nf-core/mag workflow. Because we wanted to stratify the analysis by taxonomy, we also provided a table with the GTDB taxonomy of each MAG, which was also generated by nf-core/mag. As for the genes, we extracted all the genes in KEGG’s “Assimilatory sulfate reduction” pathway. We just had to give Thanos the KEGG ortholog IDs of the genes and the paths to the files generated during the nf-core/mag workflow.

**Figure 2:**
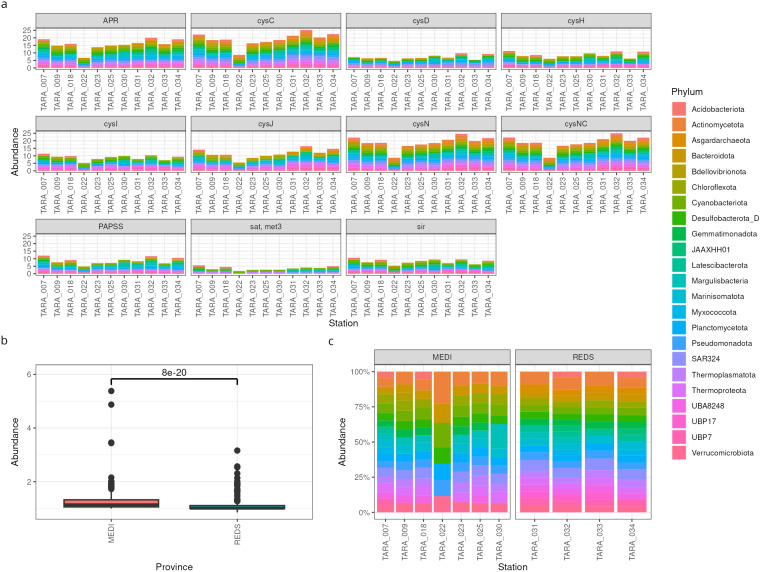
Functional profiling of MAGs. (**a**) Relative gene abundances across TARA stations, coloured by phylum. A higher bar means higher abundance. (**b**) Comparison of the relative abundance of the gene *cysCN* in two groups of samples: from the Mediterranean Sea (**left**) and from the Red Sea (**right**). (**c**) Contributions of different phyla to the total gene abundances for *cysCN*. A higher bar means higher relative abundance.

First, we explored the sulfur assimilation potential by plotting the depth score of the genes across stations (Figure 2a). This gave us a feeling for the overall differences between the stations, as well as which are the most important phyla. In this case, the abundances of sulfur genes is relatively uniform across samples, except possibly for station TARA_022, which shows lower abundances. Moreover, the contribution of phyla to sulfur assimilation also appears uniform, with no prevalent taxon. The most abundant genes are *apr*, *cysC*, *cysN*, and *cysCN*.

If we were especially interested in one enzyme, say, CysCN, we could explore it more in depth by comparing its abundance across two environments. We found that its abundance is significantly lower in the Red Sea than in the Mediterranean Sea (Figure 2b). Nevertheless, the relative contributions of the taxa are not greatly different, with the exception of station TARA_022 (Figure 2c).

### 3.2. Contigs Workflow: Prevalence of Glycolysis

**Figure 3:**
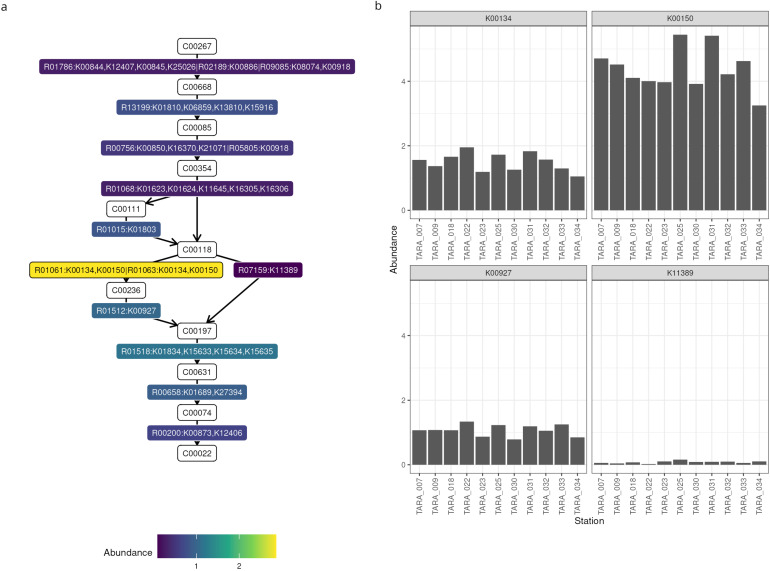
Abundances of glycolysis genes. (**a**) Annotated pathway graph. The nodes in white are the KEGG identifiers for the compounds, whereas the coloured nodes are the KEGG reactions and enzymes, coloured by their abundance. (**b**) Functional profile of four metabolic genes: *gapA* (K00134), *gapB* (K00150), *pgk* (K00927), and *gapor* (K11389).

In the second example, we examined the prevalence of glycolysis genes. As we were not interested in the taxonomies, we could use the contigs rather than the MAGs, so as to include even the unbinned DNA in the analysis. After setting up Thanos and providing it all the necessary inputs, we annotated the reaction graph of glycolysis with the depths scores computed for our samples (Figure 3a). Two reactions, namely glucose to glucose-6-phosphate performed by the glucose phosphotransferase enzyme, and glyceraldeide-3-phosphate to 3-phosphoglycerate performed by glyceraldehyde-3-phosphate dehydrogenase with ferredoxin cofactor, are almost absent. On the other hand, the enzymes phosphoglycerate kinase, catalyzing the reaction from 3-phosphoglyceroyl-phosphate to to 3-phosphoglycerate, have an average copy number of 2.8 in these samples. As this reaction can be performed by four different enzymes, we also investigated the abundance of each individual gene (Figure 3b), finding that *gapB* (K00150) and *gapA* (K00134) are the most abundant, whereas *gapor* (K11389) is virtually nonexistent.

## 4. Discussion

We developed a package to streamline a gene- or pathway-centric analysis of metagenomics data. It can analyze both contig-level data and MAG-level data within a single, general framework. The software is user-friendly and efficient, and it integrates well within the existing R ecosystem, in particular with the phyloseq and ggplot2 packages.

A notable caveat of our approach is that, even if a gene has a high DNA copy number, this does not necessarily mean that the gene will be highly expressed. Indeed, gene expression is a highly regulated process [36]: factors like the presence of nutrients, the phase of the cell cycle, or environmental stress can dramatically influence the amount of protein produced by a gene [37,38,39]. Thus, whenever possible, metagenomics data should be complemented by meta-transcriptomic or even proteomics experiments.

Furthermore, users of Thanos should keep in mind the well-known caveats of HMM-based sequence similarity searches: significant sequence similarity between two genes does not necessarily imply functional similarity [40]. For example, one of the genes could be a paralog, the result of a gene duplication. Paralogs are often subject to a lower evolutionary pressure [41], allowing their functions to drift. Pseudogenes and paralogs could introduce false positives in the results, and false negatives cannot be excluded either, but Thanos offers two ways to counter this: the possibility to adjust the significance threshold for the HMM hits and the possibility to use a custom-built HMM. A manually curated HMM profile, containing only the most specific sequences of interest, can mitigate the inclusion of pseudogenes and paralogs. A stricter sensitivity threshold would have the same effect, at the cost of potentially increasing the false negative rate as well [19].

Despite these limitations, functional profiling of metagenomic samples can offer insights into not just which bacteria populate an environment, but also what genes are there and in which average copy number (abundance). This is especially relevant, as even different strains of the same species can harbor sometimes vastly different gene portfolios, and therfore perform vastly different metabolic reactions  [42,43]. For instance, whereas the commensal strain *E. coli* K-12 is generally benign and used extensively in laboratory research, the pathogenic strain *E. coli* O157:H7 possesses additional virulence factors enabling it to cause severe foodborne illness in humans [44]. A naive taxonomic profiling approach would not distinguish these strains, as they share the same species name. However, a functional analysis facilitated by Thanos would readily highlight the presence of virulence genes in a sample.

Conventional tools such as Prokka [12,13],the EggNOG-mapper [14,15], and the KEGG mapper [17,45], operate by searching the genes within a given sample against a predefined target database. This approach inherently limits the functional detection to genes that are already documented within these reference databases, thereby excluding any potential novel or yet-to-be-characterized genes that might be present in the sample. A key advantage of Thanos over these batch-annotation tools is that, by using custom HMMs, it is possible to consider novel or uncharacterized genes, that are not annotated in sequence databases, detecting their presence and abundance in the sample.

The quantitative data provided by Thanos can be validated and understood in the context of other environmental parameters measured in the samples, such as the levels of metabolites or nutrients present. If the mere presence of a gene does not guarantee its functional activity, the corresponding correlation of its abundance with an essential and specific environmental factor related to its function could support the hypothesis that the gene’s functional potential is indeed being realized [46].

## 5. Conclusions

Our focus on functional profiling by examining genes directly rather than relying solely on taxonomic classifications has the potential to provide a more nuanced understanding of microbial community roles in various environments. Thanos is optimized for comparing functional profiles across different settings, and its applications extend beyond metabolic pathways. For instance, the package can be used to compare the abundance of pathogenic genes, antimicrobial resistance genes, or even different classes of CRISPR systems. Looking ahead, we aim to expand Thanos’s capabilities to support additional omics modalities, like metatranscriptomics, proteomics, and metabolomics, which would help get a comprehensive picture of the biological systems that are at play in a sample. Another possible application of Thanos arises in the context of metagenome-wide association studies (MWAS) [47], where it can help identify associations not between taxa and phenotypes, but between pathway abundance and phenotypes. This pathway-centric approach opens new avenues for understanding the functional implications of microbial community structures on host or environmental phenotypes. By offering a flexible and comprehensive tool for such analyses, we aspire for Thanos to facilitate novel discoveries in both environmental and biomedical research contexts.

## Data Availability

No original data were generated for this study. The metagenomics data that we analysed can be downloaded from the European Nucleotide Archive (ENA) unded the accession IDs listed in Table S1.

## Supplementary Materials

The following supporting information can be downloaded at: https://www.mdpi.com/article/10.3390/microorganisms12071264/s1, Supplementary Table S1: Samples analyzed in this paper. The table has two columns: “sample”, which lists the Experiment ID corresponding to the sample in the European Nucleotide Archive (ENA); and “group”, which indicates the co-assemblies (experiments in the same group were co-assembled). The Thanos package and the code used to produce the plots in this paper are hosted on GitHub at the url https://github.com/zhezhaozoe/thanos (accessed on 17 June 2024) under the MIT license.

## Abbreviations

The following abbreviations are used in this manuscript:

MAG	Metagenome-Assembled Genome
OTU	Operational Taxonomic Unit
HMM	Hidden Markov Model
KEGG	Kyoto Encyclopedia of Gene Elements
